# The economic case for hospital discharge services for people experiencing homelessness in England: An in‐depth analysis with different service configurations providing specialist care

**DOI:** 10.1111/hsc.14057

**Published:** 2022-10-07

**Authors:** Michela Tinelli, Raphael Wittenberg, Michelle Cornes, Robert W. Aldridge, Michael Clark, Richard Byng, Graham Foster, James Fuller, Andrew Hayward, Nigel Hewett, Alan Kilmister, Jill Manthorpe, Joanne Neale, Elizabeth Biswell, Martin Whiteford

**Affiliations:** ^1^ Care Policy and Evaluation Centre The London School of Economics and Political Science London UK; ^2^ NIHR Policy Research Unit in Health and Social Care Workforce London UK; ^3^ Institute of Health Informatics, University College London Department of Epidemiology and Public Health, Institute of Epidemiology and Health Care London UK; ^4^ Community and Primary Care Research Group, Peninsula School of Medicine University of Plymouth, ITTC Plymouth UK; ^5^ Blizard Institute, Queen Mary University of London London UK; ^6^ Pathway and the Faculty for Homeless and Inclusion Health London UK; ^7^ National Addiction Centre Institute of Psychiatry, Psychology & Neuroscience, King's College London, Addictions Sciences Building London UK; ^8^ Department of Community Nursing and Community Health Glasgow Caledonian University Glasgow UK

**Keywords:** hospital discharge, cost‐effectiveness, intermediate care, people experiencing homelessness, step‐down care

## Abstract

There are long‐standing concerns that people experiencing homelessness may not recover well if left unsupported after a hospital stay. This study reports on a study investigating the cost‐effectiveness of three different ‘in patient care coordination and discharge planning’ configurations for adults experiencing homelessness who are discharged from hospitals in England. The first configuration provided a clinical and housing in‐reach service during acute care and discharge coordination but with no ‘step‐down’ care. The second configuration provided clinical and housing in‐reach, discharge coordination and ‘step‐down’ intermediate care. The third configuration consisted of housing support workers providing in‐reach and discharge coordination as well as step‐down care. These three configurations were each compared with ‘standard care’ (control, defined as one visit by the homelessness health nurse before discharge during which patients received an information leaflet on local services). Multiple sources of data and multi‐outcome measures were adopted to assess the cost utility of hospital discharge service delivery for the NHS and broader public perspective. Details of 354 participants were collated on service delivery costs (salary, on‐costs, capital, overheads and ‘hotel’ costs, advertising and other indirect costs), the economic consequences for different public services (e.g. NHS, social care, criminal justice, housing, etc.) and health utilities (quality‐adjusted‐life‐years, QALYs). Findings were complex across the configurations, but, on the whole, there was promising evidence suggesting that, with delivery costs similar to those reported for bed‐based intermediate care, step‐down care secured better health outcomes and improved cost‐effectiveness (compared with usual care) within NICE cost‐effectiveness recommendations.

**What is known about this topic**

- About 4800 people are sleeping rough any night in England.
- Local authority funding to support single people experiencing homelessness has fallen substantially in recent years in England; many of the schemes originally funded by the Homeless Hospital Discharge Fund are now reduced in scale or have closed.
- Compared to people who are not homeless, those experiencing homelessness are likely to be discharged back onto the street (70%), attend Accident and Emergency (A&E) departments six times more frequently, be admitted three times more frequently, stay in hospital three times longer and have unscheduled hospital care eight times more frequently.

**What this paper adds**

- Specialist homeless hospital discharge schemes are potentially more effective and cost‐effective than ‘standard care’.
- Homeless hospital discharge schemes providing access to specialist intermediate care (step‐down beds) appear more cost‐effective than schemes with no access to intermediate care.
- Evidence from this study may enable the development of national and local policy guidance on the multiple factors to be considered to deliver safe, appropriate transfers of care from hospitals for people who experience homelessness.

## 1 INTRODUCTION

In England, the number of people discharged from hospitals with no fixed abode (i.e. experiencing some form of homelessness) rose by 29.8% from 6748 in 2014 to 8758 in 2018 (Marsh & Greenfield, 2019). People experiencing homelessness are affected by a disproportionate burden of chronic illnesses and, especially those rough sleeping, are among the most exposed to the risk of COVID‐19. Between April and September 2020 over 50% of services across England reported an increase in local homelessness and nearly three‐quarters (73%) an increase in demand for housing support (Boobis & Albanese, 2020). Analysis of hospital admission data shows that, between 2013/14 and 2018/19, there has been a 130% rise in the number of hospital admissions related to homelessness in England (Cream et al., 2020). Effective discharge planning can boost recovery rates and reduce hospital length of stay and unplanned readmission to a hospital (Gonçalves‐Bradley et al., 2016). Unfortunately, most hospital discharge policies and best practice guidelines are not fit to cater to patients with no fixed address, leading to inappropriate discharges and health inequities. System failures are described elsewhere (Aldridge, 2020; Jenkinson et al., 2020, 2022).

English national guidance ‘High Impact Change Model for Managing Transfers of Care’ (Local Government Association, 2020) recommends reduced assessment (for longer‐term support) at discharge. They also support the delivery of a specific set of commissioned services (including step‐down housing and clinical) to help people recover from post‐hospital stays.

In 2013, the £10 million ‘Homeless Hospital Discharge Fund’ (Department of Health and Social Care, 2013) enabled 52 local partnerships to pilot and develop a range of specialist discharge and intermediate care schemes for individuals who experienced homelessness (referred to below as ‘specialist care’; Department of Health and Social Care, 2013). However, an evaluation of the Homeless Hospital Discharge (HHD) schemes produced economic evidence of poor quality (Cornes et al., 2021). While a few publications reported that HHD schemes can be cost‐effective and also save money (see Hewett et al., 2016), their focus was exclusively on schemes that are clinically led rather than housing‐led or combined initiatives.

Between 2015 and 2019, we undertook a national evaluation of hospital discharge schemes for people who experience homelessness in England (Cornes et al., 2021). This included an economic evaluation of outcomes for 3882 users of 17 varied Homeless Hospital Discharge (HHD) schemes.

Findings from the study, which compared clinically led and housing‐led schemes and schemes with and without step‐down services, are reported elsewhere (Cornes et al., 2021). In this study, we investigate the cost‐effectiveness and cost utility of a subgroup of three ‘in patient care coordination and discharge planning’ configurations of care at three separate sites (see Supplementary Material 1) at three time points—0, 12 and 36 months. We considered different service provisions in multiple local (geographical) environments, with different accessibility to housing and other services.

## 2 AIMS AND OBJECTIVES

This study aimed to provide cost‐utility analyses comparing three different ‘in patient care coordination and discharge planning’ configurations, and ‘standard’ care, for people experiencing homelessness on discharge from the hospital to NHS and other public service agencies (such as public housing and social welfare). We also wanted to estimate the costs of these three service models and make them available to local policy decision‐makers who might be considering providing similar services. To this end, we looked at the relationship between service delivery costs (salary, on‐costs, capital, overheads and ‘hotel’ costs, advertising and other indirect costs) and the broader economic consequences for different public services (e.g. criminal justice, drug and alcohol treatment, hospitalisations and primary healthcare, housing, mental healthcare, social care, state pension costs, social security benefits, etc.), as a way of examining economic impact.

## 3 METHODS

### 3.1 Design

Two economic models were applied to address separate questions (Table 1). We considered the economic consequences for—and service delivery costs incurred by—the NHS when delivering one of three HHD service configurations and compared these with the resources used for standard care considering gains in quality‐adjusted life years (QALYs; where the benefits, in terms of length of life, are adjusted to reflect the quality of life of the person or group; NICE, 2022) (economic model 1). In addition, we investigated the cost utility of specialist integrated health and care services for people who experience homelessness from the broader public perspective (see economic model 2). In each model, we included three different ‘in patient care coordination and discharge planning’ care configurations (compared with standard care). We considered health and economic outcomes among different groups of single people experiencing homelessness receiving different service configurations and estimated the service delivery costs for each service configuration.

**TABLE 1:** Summary of economic models used in the evaluation

Type of economic model	Research question	Objective	Comparator	Measure of cost‐effectiveness	Source of data	Measure of cost	Measure of effectiveness	Perspective
Economic Model 1	What is the cost utility of SIHHC for the NHS?	Model 1 estimated the comparative cost utility of three different service configurations	We looked at the variation in effect compared with control, across sites and over time	The incremental cost utility was calculated in terms of cost per QALY gained	For the intervention groups, economic evidence was sourced from HES data; utility data were extracted from project survey/audit data collected from local sites and the literature. For the control group, both economic evidence and utility data were sourced from the literature (Hewett et al., 2016)	Service delivery (salary, on‐costs, capital, overheads and ‘hotel’ costs) and resources used (i.e. elective readmissions, emergency re‐admissions and other re‐admissions)	The measure of effectiveness was QALYs gain	The perspective adopted was NHS
Economic Model 2	What is the cost‐utility of SIHHC from the broader public perspective?	This economic model was similar to model 2, but it excluded one site (Configuration 1, clinically led/no step‐down) and did not include a control group	We compared the cost‐effectiveness of the two sites	It measured the cost utility of HHD schemes before and after their introduction in the same study site(s). Observed differences in performance were assumed to be due to the intervention (the configurations)	This economic model used audit/survey data	Service delivery (salary, on‐costs, capital, overheads and ‘hotel’ costs) plus resources used (see list reported in Supplementary Material 2, Table 2).	The measure of effectiveness was QALY gain	With this economic model, we were finally able to explore the larger public sector perspective (NHS plus criminal justice, social care, mental health, drug and alcohol services, social security benefits, housing, etc.)

Data were extracted from different sources, depending on the service configuration and type of information needed (for more details, see Table 1 and Supplementary Material 2). The various datasets were matched according to patient sociodemographic characteristics and health status (see Table 2).

**TABLE 2:** Sociodemographic characteristics and health status of study participants at baseline

	Configuration 1	Configuration 2	Configuration 3	Control (standard care)
	*n* = 206	*n* = 64	*n* = 84	*n* = 204
Source of data	Hewett et al. (2016)	Survey	Audit data	Hewett et al. (2016)
Age, mean (SD)	41.6 (12.1)	41.92 (13.1)	42.4 (12.5)	42.5 (11.3)
Male gender	168 (81.6%)	48 (75.0%)	62 (73.8%)	166 (81.4%)
Ethnicity, white (UK)	143 (69.4%)	54 (84.3%)	n/a	148 (72.5%)
Mental health condition yes	123 (59.7%)	8 (12.5%)	41 (48.8%)	113 (55.4%)
Housing status on hospital admission				
In street	82 (39.8%)	21 (32.8%)	n/a	96 (47.1%)
Unstable address	70 (34.0%)	33 (51.5%)	n/a	62 (30.4%)
In hostel	38 (18.4%)	9 (14.6%)	n/a	34 (16.6%)
Others	16 (7.8%)	1 (0.1%)	n/a	12 (5.9%)
Hospital admission in previous 12 months				
None	27 (13.1%)	31 (48.4%)	55 (65.5%)	33 (16.2%)
1	55 (26.7%)	16 (25.0%)	8 (9.5%)	52 (25.5%)
2 to 9	104 (50.5%)	13 (20.3%)	4 (4.8%)	95 (46.6%)
10 to 30	13 (6.3%)	4 (6.3%)	0 (0)	12 (5.9%)
Not given	7 (3.4%)	0 (0)	16 (19.0%)	12 (5.9%)
EQ‐5D‐5L^a^ Mean (*SD*)	0.5 (0.3)	0.5 (0.3)	0.5 (0.3)	0.5 (0.3)

a A different version of the EuroQol five‐dimensional (EQ5D) descriptive system was adopted in the survey (EQ‐5D‐3L) to the version used for the audit data and in Hewett et al. (2016; EQ‐5D‐5L). We transformed the EQ‐5D‐3L into EQ‐5D‐5L index values using the approach presented by van Hout et al. (2012) to allow for comparability across sources.

### 3.2 Study participants and inclusion criteria

Participants were hospital patients experiencing homelessness (due to being discharged with no stable accommodation to go to) seen by one of three separate specialist discharge schemes while in the hospital. Participants were eligible for the study if they were adults, 18 years or older, with at least one hospital admission.

### 3.3 Intervention groups (three separate sites, each presenting a different HHD service configuration, providing ‘specialist care’)

Three different service configurations were considered in our analyses:

- Configuration 1 comprised of a clinically led scheme providing patient in‐reach and discharge coordination, with no ‘step‐down’ service. These schemes are usually nurse or general practitioner (GP)‐led and include in‐reach (hospital ward rounds) and discharge coordination.
- Configuration 2 comprised clinical and housing in‐reach, discharge coordination and access to ‘step‐down’ intermediate care.
- Configuration 3 comprised a housing‐led scheme that primarily focused on providing accommodation to individuals with experience of homelessness on discharge from the hospital. They included a group of housing support workers providing patient in‐reach, discharge coordination and community‐based step‐down.

Full descriptions of the service configurations appear in Supplementary Material 1. Drawing upon the realist evaluation completed for the wider study (Cornes et al., 2021), we hypothesised that configuration 2 would be the preferred option in terms of both effectiveness and cost‐effectiveness because it integrated ‘specialist care’ into a clinically focused process and delivered multidisciplinary care incorporating the key elements to secure safe and timely care transfers. This seemed more comprehensive than what was offered by the other two configurations. Configuration 1 lacked direct access to step‐down intermediate care and configuration 3 had no access to a clinically led multidisciplinary team in the hospital.

### 3.4 Control group (hospital‐based clinical team providing standard care)

We originally planned two control groups: (1) people seen by Find and Treat (F&T), a community homelessness service in London, and consequently admitted to a hospital with no access to specialist integrated homeless health and care (SIHHC) scheme (Jit et al., 2011); (2) individuals experiencing homelessness who were admitted to hospital at any 1 of the 17 research sites involved in the larger study (Cornes et al., 2021). They were not in contact with the HHD scheme operating at that site. Patients for these two control groups who were not seen by an HHD scheme were generally healthier than patients in the two control groups and, as such, did not represent a suitable comparator group. We, therefore, considered a third control group (not formerly included in the protocol). As a proxy for standard care, we used the data from the control group of a randomised controlled trial (RCT) published by Hewett et al. (2016). In that RCT, patients experiencing homelessness in the control group received a visit from a homelessness health nurse and were given an information leaflet on local services. Individuals assigned to the RCT control group were comparable with those who received HHD scheme services (Table 2).

### 3.5 Sources of unit cost estimates

Sources of unit cost data are presented in Supplementary Material 2. Costs are reported in English pounds (£) based on 2017 rates. Most NHS costs were extracted from national tariffs (Netten & Curtis, 2017; see Supplementary Material 2). When not available from published sources, some estimates were obtained directly from local sites.

### 3.6 Statistical analysis

#### 3.6.1 Economic model 1: What is the cost utility of SIHHC for the NHS?

This model considered the cost utility of specialist care in terms of hospital admissions. Service delivery costs included salary, on‐costs, capital, overheads and ‘hotel’ costs. Advertising the service and other indirect central infrastructure costs were also included. Service use data for the intervention and control groups were taken from Hospital Episode Statistics (HES) and Hewett et al. (2016) respectively. QALY gains represented the measure of benefit.

The costs for each type of admission were derived from the mean resource usage of all participants within each group multiplied by the appropriate unit cost. The total costs of service use were then summed providing aggregate figures (see Supplementary Material 2). Service delivery costs for the three options were estimated on the basis of local costing data from individual study sites.

We adopted a controlled comparative approach which enabled us to measure the comparative cost‐effectiveness of three different configurations of specialist care versus standard care. For the intervention groups, hospital data were sourced from HES, utility data were taken from a retrospective longitudinal survey and audit data were gathered from study sites and contextual literature (Hewett et al., 2016). For the control group, both economic evidence and utility data were extracted from published sources (ibid).

For all participants within each group, we estimated the differences in mean 12‐month costs and outcomes. Data were presented per person experiencing homelessness. As a measure of cost‐effectiveness, we calculated incremental cost‐effectiveness ratios (ICERs) by computing the difference between intervention and control groups in mean cost and then dividing it by the difference in mean health outcome. This provided a measure of the incremental cost to be invested per QALY gained.

Hewett et al. (2016) used standardised health‐related quality‐of‐life questionnaire (EuroQol 5 Dimension, EQ‐5D; https://euroqol.org/) at both baseline and 12‐month follow‐up. But they did not provide any (retrospective) baseline data for the length of stay and use of healthcare resources. In this study, we replicated the regression model adopted by Hewett et al. (2016) and we adjusted differences in EQ‐5D score at follow‐up for variation in patients' characteristics, including gender and age, and for differences in EQ‐5D at baseline.

UK tariff was applied to calculate utility values (Kind et al., 1999; van Hout et al., 2012). QALYs were then calculated using the area under the curve method (Richardson & Manca, 2004). Different versions of the EQ‐5D descriptive system were adopted across data sources. To allow comparability between intervention and control groups, we transformed EQ‐5D‐3L values collected for configuration 2 into EQ‐5D‐5L values, as per configurations 1 and 3 and the control group (van Hout et al., 2012).

To establish if a particular configuration of specialist care offers value for money, the ICER was compared to the cost‐effectiveness threshold used by NICE (£20,000–£30,000 per QALY). Interventions are considered cost‐effective if their ICER falls below the NICE's threshold.

#### 3.6.2 Economic model 2: What is the cost utility of SIHHC from the broader public perspective?

We compared the cost‐effectiveness of two separate ‘in patient care coordination and discharge planning’ care configurations (2 and 3) ‘before and after’ their introduction. It allowed us to broaden the perspective to economic impacts beyond NHS hospital admissions (e.g. A&E visits, hospitalisations, hospital outpatient attendances and GP visits) to include criminal justice, drug/alcohol treatments, housing, mental health services, social security benefits, social care services and state pensions. In this model, we did not use a control group because Hewett et al. (2016) did not capture the use of community resources data. We assumed any observed differences in performance between configurations to be caused by the intervention. NHS and social care costs attached to the delivery of the intervention costs were also included. Both cost and utility data were taken from the project survey and local audit data gathered from research sites as well as the literature.

This economic model adopted a similar approach to the Making Every Adult Matter (MEAM) evaluation (Flatau & Zaretzky, 2008). The purpose of the MEAM pilot programmes was to understand the economic impact of specialist care provision on a cohort of people with multiple and complex needs including individuals with experience of homelessness. The MEAM pilot programmes developed and tested a robust methodology for assessing the economic impact of specialist care provision on service use for the public system.

The utility was measured in terms of QALY gain. Annual cost estimates were calculated from NHS (limited to hospitalisations) and larger public sector perspectives. We included resource use and costs for 1 year follow‐up and a 3‐year projection.

### 3.7 Time horizon

We considered a 12‐month time horizon for both costs and benefits. We also explored a 3‐year scenario with the assumption the person keeps experiencing homelessness and costs and benefits are constant over time. For the 3‐year projection model, we considered a 3.5% discount rate.

### 3.8 Sensitivity analyses

One‐way sensitivity analyses were undertaken to assess the impact on the base case results of different cost measures, a shift in costs from non‐elective to elective readmissions and also a change in relative effectiveness measures and a longer time frame. A probabilistic sensitivity analysis allowed us to quantify the robustness of the output of the analysis in relation to uncertainty in the model inputs (see Supplementary Material 3 for more details).

## 4 RESULTS

### 4.1 Sample characteristics

Drawing on the different data sources, we accessed a total of 354 people with complete information on the use of healthcare resources (HES data) and health outcomes (EQ‐5D) at baseline and 248 people at 12 months. Participants' sociodemographic data are presented in Table 2. Further detail and discussions of the challenges experienced when collecting survey data are presented elsewhere (Cornes et al., 2021).

### 4.2 Economic model 1: What is the cost utility of SIHHC for the NHS?

#### 4.2.1 Cost per patient for each readmission

**FIGURE 1:**
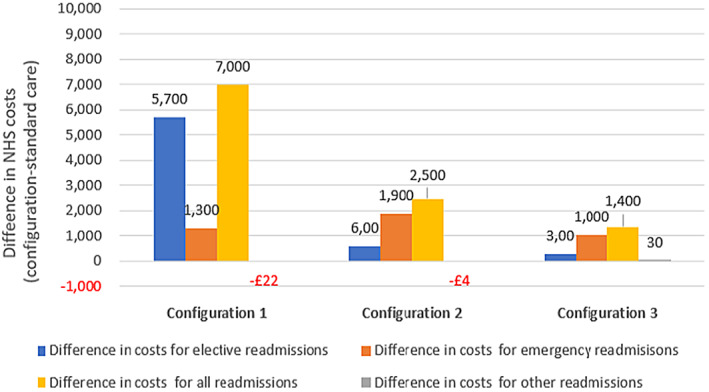
Economic model 1: Difference in annual NHS costs per patient across three out‐of‐hospital care configurations (vs. standard care). Note: Difference in annual NHS costs per patient between configuration and standard care. Configuration 1 (clinically led only), configuration 2 (clinically led/residential step‐down) and configuration 3 (housing‐led/community step‐down).

Figure 1 presents the difference in annual NHS costs per patient across three out‐of‐hospital care configurations (vs. standard care). Taking into account all readmissions, configuration 1 (clinically‐led/no step‐down) and configuration 2 (clinically‐led/residential step‐down) presented substantially greater costs than configuration 3 (housing‐led/community step‐down) (£7000 vs. £2500 vs. £1.400, *p* < 0.01). Configuration 1 had the highest costs for elective readmissions, followed by configurations 2 and 3 (£5700 vs. £600 vs. £300, *p* < 0.01). Configuration 2 had the highest costs for emergency readmissions, followed by configurations 1 and 3 (£1900 vs. £1300 vs. £1000, *p* < 0.01). Configuration 3 had the lowest costs for all types of hospital stays.

#### 4.2.2 QALY gains per patient

**FIGURE 2:**
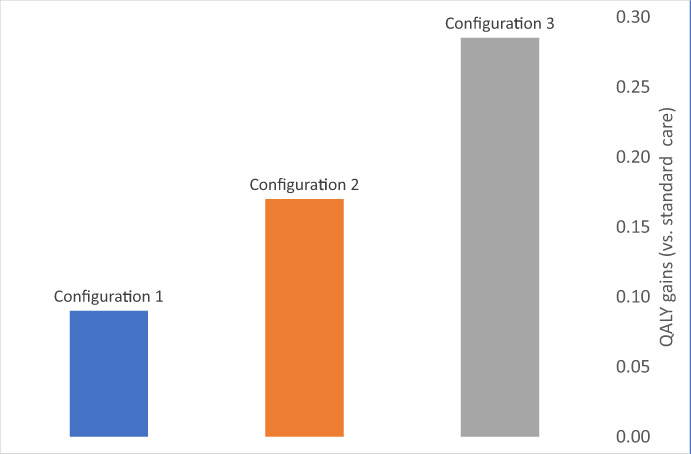
Economic model 1: Difference in annual QALY outcomes per patient (vs. standard care). Note: We report the difference in annual QALY per patient between configuration and standard care. For each configuration, the difference is positive and therefore indicates the number of QALYs gained compared with standard care. Intervention: QALY data for configuration 1 were extracted from Hewett et al. (2016), whereas local data were used for configurations 2 and 3. QALY data for standard care were extracted from Hewett et al. (2016). Configuration 1 (clinically led only), configuration 2 (clinically led/residential step‐down) and configuration 3 (housing‐led/community step‐down).

All three configurations present better QALY outcomes compared with standard care (Figure 2). Configuration 3 presents the highest number of QALY gains (compared with standard care) followed by configuration 2 and then configuration 1 (0.29 vs. 0.17 vs. 0.09). *Service delivery costs* (Table 3). From the perspective of the public provider, the average costs per individual experiencing homelessness for configurations 2 and 3 were £6100 and £2200 respectively. When considering the NHS‐related costs only, the average costs per individual experiencing homelessness were lower for both groups, but more dramatically for configuration 2 than for configuration 3. For configuration 1 (with no access to a step‐down service), service delivery costs included £800 (for the NHS budget) and were well under the mean costs per individual experiencing homelessness reported for the other configurations.

**TABLE 3:** Annual service delivery costs (year 2017)

Items	Configuration 1	Configuration 2	Configuration 3
	(Provision for 206 users from the RCT)	(Provision for 80 users)	(Provision for 88 users)
1. Paid staff – total	£123,500	£264,000	£122,500
2. Staff training, travel and subsistence expenses (e.g. for outreach activities)	£12,300	£3500	£10,000
3. Overhead costs – non‐staff	£22,400	£105,200	£7700
4. Overhead costs – staff	£10,300	£1500	£3200
5. Capital overheads	£2600	£58,900	£29,300
6. Hotel costs (e.g. food and cleaning)	£0	£13,400	£0
7. Advertising of the service	£0	£0	£300
8. Other indirect central infrastructure costs	n/a	£40,800	£6000
NHS perspective (see items 1–5 above)			
Total yearly costs, 2017	£171,000	£433,200	£172,600
Average cost per patient experiencing homelessness^a^	£800	£5400	£2000
Public sector perspective (see items 1–8 above)			
Total yearly costs, 2017	n/a	£487,400	£179,000
Average cost per patient experiencing homelessness^a^	n/a	£6100	£2000

*Note*: NHS perspective: intervention costs incurred by NHS covering items 1–5 (see Hewett et al., 2016). Public sector perspective: intervention costs incurred by the broader public sector (including NHS, social care and housing) covering items 1–8.

^a^ We relied on expert opinion to calculate an average estimate per patient experiencing homelessness assuming that an equal amount of resources was allocated to each patient they provided care for in the year.

#### 4.2.3 The cost‐effectiveness of different ‘in patient care coordination and discharge planning’ care configurations (compared with standard care)

If we consider service delivery costs (together with all re‐admissions costs), the ICER varied among £72,700 (configuration 1), £39,500 (configuration 2) and £8000 (configuration 3).

#### 4.2.4 Sensitivity analysis

**FIGURE 3:**
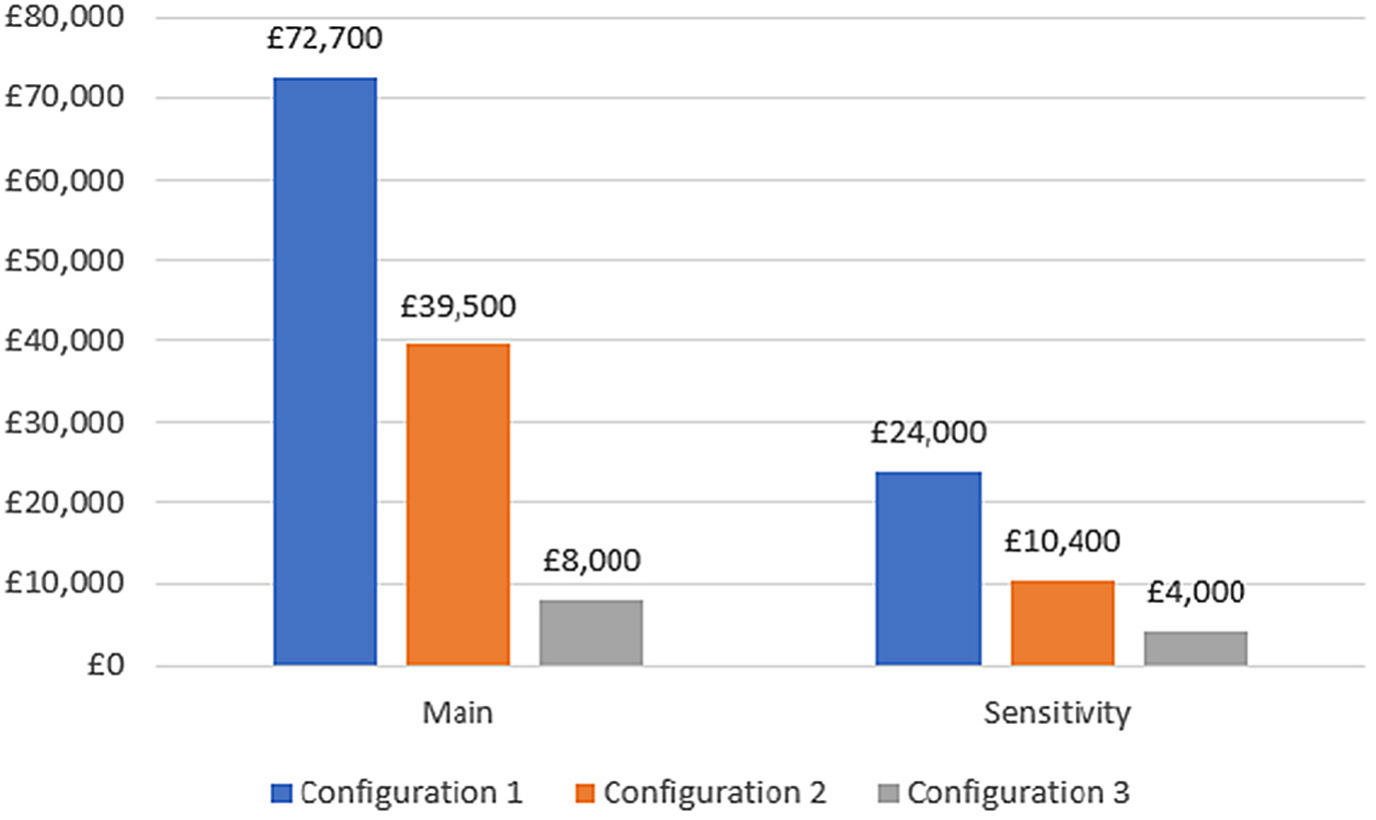
Economic model 1: Additional NHS costs to be invested per additional QALY gained (vs. standard care; incremental cost‐effectiveness ratio). Note: Main analysis: NHS resources include costs for all re‐admissions (with service delivery costs); Sensitivity analysis: NHS resources include costs for non‐elective (emergency) re‐admissions (with service delivery costs). Utility data used here are reported in Figure 2. The cost‐effectiveness analyses reported positive costs and positive effects, and trade‐offs between costs and effects were considered. This represents the situation where intervention may be cost‐effective compared to standard care, and the value at which the incremental cost‐effectiveness ratio (ICER) is considered good value for money is provided by NICE (under the threshold of £20,000 to £30,000 per QALY). Configuration 1 (clinically led only), configuration 2 (clinically led/residential step‐down) and configuration 3 (housing‐led/community step‐down).

To test the impact of the intervention, we omitted non‐elective (emergency) costs and considered elective readmissions (as an indicator of appropriate care pathway treatment). All interventions were cost‐effective according to NICE's recommendations (see Figure 3—sensitivity). The ICERs decreased to £24,000 (configuration 1), £10,400 (configuration 2) and £4000 (configuration 3).

#### 4.2.5 Other sensitivity analyses

Additional sensitivity analyses considered variations in overall costs or effectiveness for the control group, as well as for the 3‐year follow‐up. They confirmed the results reported in Figure 3. The main results from the probabilistic sensitivity analyses showed that configuration 1 has a null probability of being cost‐effective, whereas configurations 2 and 3 presented some probability of being cost‐effective (80% at a threshold of £30,000; 100% at a threshold of £8000, respectively).

### 4.3 Economic model 2: What is the cost utility of SIHHC from the broader public perspective?

For this model, we used linked HES data and survey data to assess the cost utility of different ‘in patient care coordination and discharge planning’ care configurations considering the NHS and the wider public perspective. As reported above, this analysis is limited to configurations 2 and 3 because configuration 1 did not capture the use of community resources data.

**FIGURE 4:**
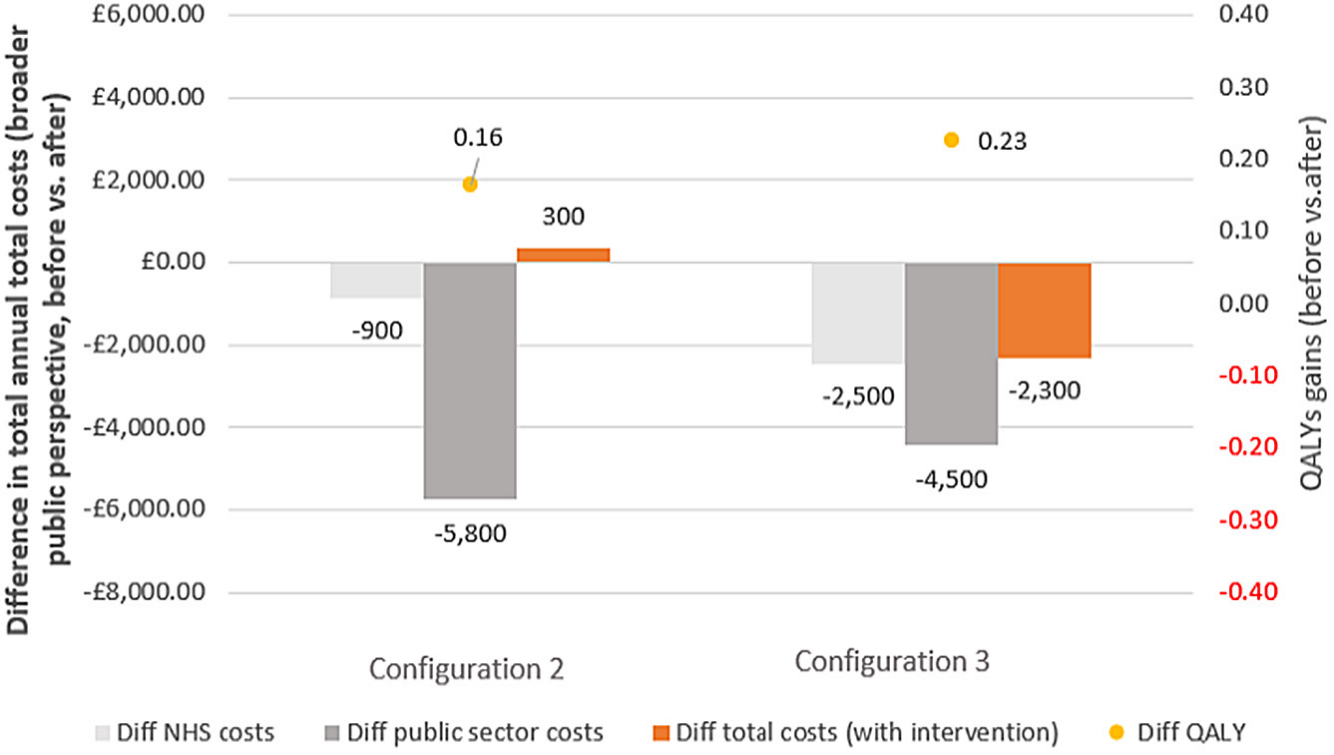
Economic model 2: Difference in total annual total costs (NHS and broader public perspective) and QALY outcomes per patient (comparison before and after). NHS costs data covered A&E visits, hospitalisations, hospital outpatient attendances, GP visits and are comparable across sites. Public sector costs covered NHS costs above plus mental health services, drug and alcohol treatment services, housing costs, criminal justice (configuration 2 only), social care costs, social benefits (configuration 2 only) and state pension (configuration 2 only; see Supplementary Material 2). Total costs with intervention: Public sector and total cost estimates are not comparable across configurations as the type of data available on the use of resources was inconsistent across local sites. Configuration 2 (clinically led/residential step‐down) and configuration 3 (housing‐led/community step‐down). Please note that this analysis is limited to configurations 2 and 3 because configuration 1 did not capture the use of community resources data.

When looking at the difference in costs for healthcare resources (A&E visits, hospitalisations, hospital outpatient attendances and GP visits), we reported an annual cost saving (between £900 for configuration 2 and £2500 for configuration 3 [Figure 4]). For both configurations, the cost saving (compared with baseline) was greater when considering the broader public provider perspective (£5800 and £4500, respectively). The impact of both configurations on public cost saving was greater on services beyond the NHS. When considering total healthcare costs (all the above plus service delivery costs), there was a cost saving of £2300 for configuration 3, whereas for configuration 2, there was an increase in NHS costs of £300. QALY gains increased to 0.16 for configuration 2 and 0.23 for configuration 3 (Figure 3).

#### 4.3.1 The cost‐effectiveness of different ‘in patient care coordination and discharge planning’ care configurations (comparing 1 year before and 1 year after their introduction)

**FIGURE 5:**
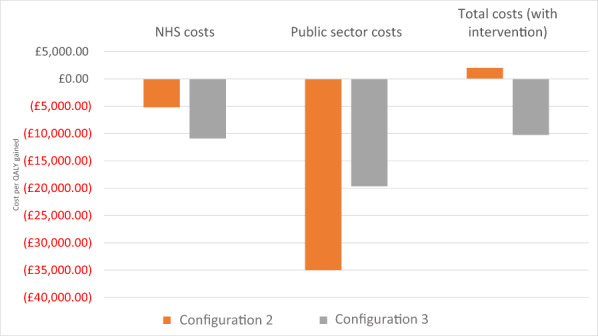
Economic model 2: ICER, cost per QALY gained (project local survey sites, comparison before and after). Note: All ICERs indicate favourable options according to NICE thresholds. A positive ICER indicates that the configuration is cost‐effective (C > 0, E > 0), whereas a negative ICER (C < 0, E > 0) indicates that the configuration is both cost‐effective and cost saving. ICER estimates based on the public sector and total cost estimates (total costs with intervention) are not comparable as the type of data available on the use of resources was inconsistent across sites. Configuration 2 (clinically led/residential step‐down) and configuration 3 (housing‐led/community step‐down). Please note that this analysis is limited to configurations 2 and 3 because configuration 1 did not capture the use of community resources data.

The cost‐effectiveness analyses (total costs for the broader public sector perspective with service delivery costs) for configuration 3 presented cost savings (compared with usual care) and positive effects (C < 0 & E > 0; Figure 5). For configuration 2, we found positive costs (more resources to be invested compared with usual care) and positive effects (C > 0 & E > 0; £2000 cost per QALY). Both configurations were cost‐effective according to NICE recommendations. In addition, configuration 3 was also cost saving.

#### 4.3.2 Sensitivity analyses

Deterministic scenario analyses (Supplementary Material 3) produced similar results to those already reported above. Probabilistic sensitivity analyses showed that both configurations have a 100% probability of being cost‐effective according to NICE recommendations.

## 5 DISCUSSION

Across the different modelling exercises (summarised in Table 4), the findings from our economic models confirm that ‘specialist care’ secures better health outcomes and is more cost‐effective than standard care. For the NHS (model 1), all three configurations of specialist homeless discharge care appear cost‐effective in terms of NICE thresholds for cost per QALY. For the wider public provider perspective (model 2), configurations 2 and 3 are within NICE cost‐effectiveness recommendations. Configuration 1 was not considered in this analysis because of the lack of service cost data.

**TABLE 4:** Summary of results from all economic models

	Configuration 1	Configuration 2	Configuration 3
**Economic model 1: What is the cost utility of SIHHC for the NHS?**			
… Compared with the control	[*economic outcome, all re‐admission costs*]		
	Not cost‐effective	Cost‐effective	Cost‐effective
	[*economic outcome, non‐elective re‐admission costs*]		
	Cost‐effective	Cost‐effective	Cost‐effective
… Across sites	Conf.3 > conf.2 > conf.1 [*all re‐admission costs*]		
	Conf.3 > conf.1 > conf.2 [*non‐elective re‐admission costs*]		
… Across time	No change in 3 years for all		
**Economic model 2: What is the cost utility of SIHHC for the broader public perspective?**			
… Compared with the control	N/a	Cost‐effective	Cost‐effective & cost saving
… Across sites	N/a	Conf.3 > conf.2	
… Across time	N/a	No change in 3 years for all	

*Note*: Configuration 1 (clinically led only – no data on broader public perspective), configuration 2 (clinically led/residential step‐down) and configuration 3 (housing‐led/community step‐down).

*Service delivery costs* for configuration 1 were funded by the NHS only (£800 annual costs per individual) and were well under the average NHS costs per patient experiencing homelessness reported for the other two configurations (£5400 and £2000). When including social care budgets, the average cost per patient experiencing homelessness for configuration 2 was three times as much as reported for configuration 3.

In addition, we provide evidence to support the case for ‘step‐down’ intermediate care. In economic terms, the two configurations that provide ‘step‐down’ consistently outperformed the configuration without access to ‘step‐down’. Based on our realist hypothesis (Cornes et al., 2021), we expected that configuration 2 would be the most cost‐effective as it incorporated more of the important ‘jigsaw pieces’ compared with other configurations. However, on nearly every metric, configuration 3 performed better. It presented a uni‐professional housing‐led site with a single HHD scheme in operation, with no access to specialist clinically led support. Our finding questions some assumptions about the cost‐effectiveness of clinically led multi‐disciplinary teams compared with other specialist configurations. With our modelling, we were able, somehow, to control for context in that both configurations 2 and 3 presented similar levels of rough sleeping and were located in geographically similar areas. However, we were not capable of controlling for case mix and the possibility that configuration 2 was able to treat more ‘complex patients’ because it had access to both clinical support and residential step‐down beds (with associated increased costs for these beds, e.g. ‘hotel’ costs). If this was the case, it may explain why clinically led configuration 2 looked less cost‐effective than the housing‐led service in configuration 3. Evidence that this may be occurring is that configuration 3 presented much lower costs related to emergency and elective readmissions, potentially suggesting a healthier cohort of patients. Configuration 1 also generated many more planned re‐admissions which increased their subsequent bed days and reduced their apparent cost‐effectiveness; this could also be the result of clinical advocacy improving follow‐up for a more complex patient group. Alternatively, it may be that clinically led configurations are more expensive but they might still lead to better healthcare and outcomes in a longer timeframe than we had here.

### 5.1 Potential methodological limitations

The findings showed the potential of well‐integrated HHD schemes catering to both continuous clinical and housing‐related support, in the hospital and ‘step‐down’ intermediate care. The choice of the three HHD schemes for this analysis was not planned a priori. Although the HHD schemes providing access to the outcome data were not purposively selected, they still allowed to compare three very different ‘in patient care coordination and discharge planning’ configurations. Of course, the comparisons were limited due to the fact that the data sources were not standardised for all sites and geographical environments varied in a way that could not be controlled. More challenges (e.g. in relation to the perspective adopted, comparative analyses and control group, access to data, service delivery, percentage of patients having access to step‐down care, model assumptions, etc.) encountered in this study are commented on in Cornes et al. (2021) where we also set out implications for future research. The fact that configuration 1 was in an urban environment with a higher workload and dealing with a higher number of people with more complex needs than other sites might have had a negative effect on the final outcomes.

### 5.2 Comparison of findings with the literature

The economic impact of homelessness on the public sector and society is well recognised (Crisis, 2020). Individuals who experience homelessness for more than 3 months cost on average £4300 per person to the NHS, £2100 per person for mental health services and £12,000 per person to the criminal justice system on a yearly basis (Pleace & Culhane, 2016). Such estimates are comparable with our baseline data from economic model 2 site 2, covering similar cost categories. However, evidence on the effectiveness and cost‐effectiveness of homeless discharge services is sparse. The most robust evidence is limited to the cost‐effectiveness of clinically led schemes (Cornes et al., 2021). Our NHS calculations for configuration 2 (clinically led/residential step‐down service, £5400 per user each year) are aligned with the delivery costs for bed‐based intermediate care reported by the National Audit of Intermediate Care (2018; £5500).

### 5.3 Implications for policy

This study's findings contributed to the publication of a toolkit designed to help providers and commissioners when developing out‐of‐hospital care to make sure we consistently deliver safe and timely transfers of care for individuals who experience homelessness (Cornes et al., 2019). It supplies data on the effectiveness and cost‐effectiveness of different models and configurations of ‘specialist care’ services piloted through the HHDF, and a ‘road map’ or checklist of the complex set of factors that decision‐makers should consider to shape services as inclusive as possible. Also, the checklist can help to map those areas where provision is weaker and act as a sensitivity tool for the ‘High Impact Change Model for Improving Transfers of Care Between Hospital and Home’ (Local Government Association;, 2020).

## CONFLICT OF INTEREST

Nothing to declare.

## AUTHORS' CONTRIBUTION

MT, RW and MC conceptualised and designed the study. MT analysed the data. All authors contributed to the interpretation of the results. MT, RW and MC contributed to the first draft writing. All authors reviewed and edited the manuscript. All authors have read and agreed to the final version of the manuscript.

## FUNDING INFORMATION

NIHR Health Services and Delivery Research Programme. HS&DR – 13/156/10.

## ETHICS STATEMENT

Health Research Authority Research Ethics Committee granted approval for the study (REC 16/EE/0018). Local research approvals were granted before the collection of data at each of the 17 SIHHC sites. In addition, we obtained approval from the Secretary of State for Health through the CAG (reference 16/Confidentiality Advisory Group [CAG]/0021). This allowed us to get access to identifiable patient data without individual consent.

## Supporting information


Supplementary Material S1

## Data Availability

Data that supports the findings of this study are available in the supplementary material of this article.
