# Supplementary material for: The economic case for hospital discharge services for people experiencing homelessness in England: An in‐depth analysis with different service configurations providing specialist care
Source: Health Soc Care Community. 2022 Oct 7;30(6):e6194–205. doi: 10.1111/hsc.14057 (PMC10092708; doi:10.1111/hsc.14057)
Supplement: Supplementary file 1 — Supplementary Material S1 [file HSC-30-e6194-s001.docx]

**Supplementary material**

## Supplementary material 1: List of specialist integrated homeless health and care (SIHHC) configurations

**Configuration 1 (clinically-led scheme offering patient in reach & discharge coordination - with no step-down):** A multidisciplinary ‘clinically-led’ [homeless healthcare] team (offering patient in-reach and specialist discharge coordination) with no direct access to ‘step-down’ intermediate care. Substantive support for the patient usually ends at the point of exit from the acute sector. Context: Two examples of this configuration are considered, one situated in a city, one in a seaside resort in Southern England. The regions in which these places are situated have the highest percentages of people sleeping rough in England (27% and 20% respectively) (MCHLG, 2018). Qualitative reports suggest severe shortages of housing and support services. Many patients will not have local connections to the area. Data Source: HES Data and EQ-5D (from Hewett et al.’s 2016 RCT).

**Configuration 2 (clinically led scheme offering patient in reach & discharge coordination (with residential step-down):** In this site, two HHD schemes work together in an integrated way. This configuration comprises a clinically-led [homeless health care] team offering patient in-reach and discharge coordination. This site also has access to a 14-bed residential facility offering step-down intermediate care providing support to patients for up to12 weeks post-discharge. The hospital-based homeless health care team provides ‘clinical in-reach’ into the residential intermediate care facility ensuring continuity of multidisciplinary support for patients. Context: Old industrial city, North England (Yorkshire). This region has 5% of the people sleeping rough in England (MCHLG, 2018). Qualitative reports suggest a good supply of housing and housing-related support services. Data Source: HES Data and EQ-5D (from survey data).

**Configuration 3 (clinically-led scheme offering patient in reach & discharge coordination (with community based step-down):** In this site, there is a single housing-led HHD scheme in which a small team of housing support workers visit the hospital to provide patient-in reach and discharge coordination. The housing workers then continue to support the patient in the community (offering floating support) until longer-term services are in place and working well (community ‘step-down’ intermediate care). This is usually for around 12 weeks post-discharge. The scheme is uniprofessional and does not have access to a multidisciplinary clinically-led homeless health care team based at the hospital. Context: Old industrial city/ Midlands. This region contains 8% of the people sleeping rough in England (MCHLG, 2018). This HHD is run by a housing association with direct access to a good supply of accommodation. The city has, however, experienced severe cuts to housing-related support services. Data Source: HES data and EQ-5D data (from audit data).

## Supplementary material 2: Source of data

**Collection of resource use data**

***Historical cohort data****.* First, patient data were collected at three different sites (representing the three service configurations). Eligible participants were adults over 18 years of age with one or more hospital admissions between 1 November 2013 and 30 November 2016. This included identifying demographic data for relevant hospital inpatients during the study period. Following that, patient-level data on the use of NHS resource (in terms of elective readmissions, emergency readmissions and other readmissions) were extracted from Hospital Episode Statistics (HES). The two databases above were linked following a protocol presented elsewhere (Blackburn et al., 2017). Aggregate level information used to inform our analyses for economic models 1 and 2 are presented in Supplementary material 2, table 1. Due to the lack of national datasets, we were unable to include data on the care provided in primary care and community. As a result, we were unable to examine any contribution to the health and care of the individuals from other service providers outside of NHS hospital care. In addition, the historical cohort data did not cover any information on the health-related utility that patients experiencing homelessness attach to their quality of life.

***Survey and audit data.*** Following the limitations of the historical cohort data, a pilot survey was run of 70 individual participants (all users enrolled in the local HHD scheme between 1 April 2015-31 December 2015 from configuration 3), in order to collect data that would allow to determine the broader public sector resources (e.g. in terms of hospital and primary care, social care, criminal justice, housing, etc) that participants were likely to use. On the basis of this, it was decided to collect details of the resource use items listed in Supplementary material 2 from a number of sites (se historical cohorts above). This was possible only for configurations 2 (using survey) and 3 (from audit data) and such evidence was used to inform model 2 (broadening the views beyond NHS hospital care).

The survey (and the audit data) allowed also to capture information on health-related utility estimates derived from EuroQol five-dimensional (EQ-5D) questionnaire. Individuals were assessed at baseline (data was collected shortly before discharge from hospital) and 3 months after discharge, from Oct 2016- Jan 2018. Audit data were collected from July 2014 – December 2016. The follow-up questionnaire (audit assessment form) was designed to ascertain data on the retrospective use of resources in the previous 3 months and monitor health outcomes.

***Published evidence from a RCT study (Hewett et al., 2016)***. Participants to the RCT were recruited using similar procedures as for the survey and audit data. They were interviewed by staff in the HHD schemes at baseline (whilst still in hospital, after they had been determined to be ‘medically stable’), and then, as far as was possible, three months later after discharge form hospital. Questionnaires were used for the collection of data encompassing similar dimensions (to the survey and data audit sources), being sociodemographic, use of resources, and health status.

**Source of unit cost data**

Sources of unit cost estimates are shown in Supplementary material 2, table 2. All costs are in UK pounds (£) and are 2017 figures (the period over which participants were studied and the resources were used). Most of the NHS resources costs were taken from the nationally recognised figures of Netten & Curtis (2017). To maximise the generalisability of the study findings, we aimed, as far as was possible, to use validated costs which represented national figures. Social Services inputs are less straightforward to cost (Netten & Curtis 2017) and some estimates were obtained directly from local sites.

**Collection of effectiveness estimates (economic model 1)**

The primary outcome chosen for the cost-effectiveness analyses was the cumulative duration of hospital stay (number of bed days after the index admission). This followed published literature (Hewett et al., 2016). Effectiveness estimates for the three service configurations were sourced from the historical cohort data, whereas the RCT study published by Hewett et al. (2016) provided data on the control group (see Supplementary material 1).

**Collection of utility estimates (economic models 2 and 3)**

Utility estimates were derived from EQ-5D data sources for the different comparative groups. In our study we were able to collect EQ-5D data for configurations 2 (survey) and 3 (audit data). In addition, utility values per patient for configuration 1 (and the control) were sourced from Hewett et al. (2016) ‘advanced care’ (and ‘standard care’ groups, respectively).

### Supplementary material 2, table 1: NHS resource items, number of completed admissions, bed days and QALY estimates per patients in one year after discharge by configuration and control group (economic models 1 and 2)

| Configuration | Source of data | No. Of elective readmissions per patient | | No. Of  emergency re-admissions per patient | | No. Of other re-admissions per patient | | No. Of bed days per patient | | No. Of QALY per patient | | No. Of patients at index discharge |
| --- | --- | --- | --- | --- | --- | --- | --- | --- | --- | --- | --- | --- |
|  |  | Baseline | follow up | Baseline | follow up | Baseline | follow up | Baseline | follow up | Baseline | follow up |  |
|  |  |  | Mean (SD) |  | Mean (SD) |  | Mean (SD) |  | Mean (SD) |  | Mean (SD) |  |
| Conf. 1 | HES DATA | n/a | 1.62 (12.13) | n/a | 2.47 (4.5) | n/a | 0.12 (0.63) | n/a | 18.24 (34.48) | n/a | [0.56 (0.3),  Hewett (2016)] | N=703  [N=269] |
| Conf. 2 | HES DATA | n/a | 0.31 (0.81) | n/a | 2.99 (5.38) | n/a | 0.13 (0.8) | n/a | 15.90 (32.34) | n/a | [0.64 0.22,  project survey data] | N=340  [N=50] |
| Conf. 3 | HES DATA | n/a | 0.23 (1.23) | n/a | 2.21 (3.9) | n/a | 0.15 (0.47) | n/a | 0.9 (1.7) | n/a | [0.75 (0.11),  project audit data] | N=188  [N=70] |

### Supplementary material 2, table 2: Resource items and their unit costs

| **Type of cost** | **(configurations 1 and 2)** | **(configuration 3)** | **Unit cost (2017)** | **Source of unit costs** |
| --- | --- | --- | --- | --- |
| **Healthcare costs** | Hospital Admissions (average) | Hospital Admissions (average) | £1,783 | PSSRU 2017 unit costs of health and social care |
|  | Elective inpatient stays | Elective inpatient stays | £3903 | PSSRU 2017 unit costs of health and social care |
|  | Emergency admission* | Emergency admission* | £1,074 | PSSRU 2017 unit costs of health and social care |
|  | Visits to A&E | Visits to A&E | £157 | PSSRU 2017 unit costs of health and social care |
|  | Hospital outpatient attendances | Hospital outpatient attendances | £137 | PSSRU 2017 unit costs of health and social care |
|  | GP visits (GP contact per patient contact lasting 9.22 minutes) | GP visits (GP contact per patient contact lasting 9.22 minutes) | £38 | PSSRU 2017 unit costs of health and social care |
|  |  | 999 Ambulance (average all callouts) | 205 | PSSRU 2017 unit costs of health and social care |
| **Mental health care costs** | Admission to a mental health hospital (Mental health care clusters per day) | n/a | £404 | PSSRU 2017 unit costs of health and social care |
|  | contact with Mental health community provision (Mental health specialist teams (per care contact)) | n/a | £172 | PSSRU 2017 unit costs of health and social care |
|  | Stay in a local authority care home for people with mental health problems [average duration 4*12 weeks] | n/a | £786 | PSSRU 2017 unit costs of health and social care |
|  | Access to local authority social services day-care for people with mental health problems | n/a | £35 per client attendance | PSSRU 2017 unit costs of health and social care |
|  | n/a | Mental health services for the 1 year (average based on Derby data) | £2,670 | PSSRU 2017 unit costs of health and social care |
| **Drug and alcohol treatment costs** | Substitute prescriptions (methadone) | n/a | £55 a week | Gossop 2015 |
|  | detox and rehab centre stay | n/a | Average £417 | PSSRU 2017 unit costs of health and social care |
|  | one-to-one contacts with a drug/alcohol treatment team | n/a | Average £125 | PSSRU 2017 unit costs of health and social care |
|  | Group session contacts with a drug/alcohol treatment team | n/a | Average £16 | PSSRU 2017 unit costs of health and social care |
|  | n/a | Drug and alcohol treatment services for the 1 year (average based on Derby data) | £1,061 | PSSRU 2017 unit costs of health and social care |
| **Housing costs** | Rough sleeping | Rough sleeping | £7,900 | Ashton and Hempenstall (2009) |
|  | Direct access hostel (night) | n/a | £48 | Ashton and Hempenstall (2009) |
|  | The second stage supported accommodation (Semi-independent accommodation) | n/a | £27 per night | MEAM 2014 |
|  | Own social tenancy | n/a | £69 per week | Average from MEAM 2014 |
|  | Own private rented sector tenancy | n/a | £100 per week | Average from MEAM 2014 |
|  | Room in shared private rented sector property | n/a | £65 per week | Average from MEAM 2014 |
|  |  | Homelessness investigation and decision | £6639 | Shelter’s “Value for Money in Housing Options and Homelessness” Report (2010) |
| **Criminal justice costs** | Arrest by police | n/a | £2,130 | Think Family 2010 |
|  | Other police contact | n/a | £17 | Winsor (2011) https://www.gov.uk/police-pay-winsor-review |
|  | Magistrate court attendance | n/a | £995 | Average from MEAM 2014 |
|  | Crown court attendance | n/a | £11,245 | Average from MEAM 2014 |
|  | Nights in prison/Nights in police custody | n/a | £75 | Average from MEAM 2014 |
| **Social care costs** | Comprehensive Clinical Assessment | n/a | £55 | PSSRU 2017 unit costs of health and social care |
|  | n/a | OT assessment | £35 | PSSRU 2017 unit costs of health and social care |
|  | Visit with social worker | Visit with a social worker | £55 | PSSRU 2017 unit costs of health and social care |
|  | Night in residential care | Night in residential care | £543 per week | PSSRU 2017 unit costs of health and social care |
| **Social benefits** | Employment Support Allowance | n/a | £73.10 per week | Benefits entitlement |
|  |  |  |  | Gov.uk |
|  | Personal Independence Payment (PIP) | n/a | £57.30 per week | [Benefits entitlement](https://www.gov.uk/browse/benefits/entitlement) |
|  | (Daily living - standard rate) |  |  | Gov.uk |
|  | Disability Living Allowance (DLA) for adults | n/a | £57.30 per week | [Benefits entitlement](https://www.gov.uk/browse/benefits/entitlement) |
|  | [Frequent help or constant supervision during the day, supervision at night or someone to help you while on dialysis] |  |  | Gov.uk |
|  | Universal credit | n/a | £317.82 per month | [Benefits entitlement](https://www.gov.uk/browse/benefits/entitlement) |
|  |  |  |  | Gov.uk |
| **State pension** |  | n/a | £125.95 per week for those reaching SPA before April 2016 (SPA = state pension age) | [Benefits entitlement](https://www.gov.uk/browse/benefits/entitlement) |
|  |  |  |  | Gov.uk |
| **Service delivery costs** | Conf.2 site delivery costs were calculated using costing information collected from the site | Service delivery costs were calculated using costing information collected from the site | See table 2, main text | Hewett et al. (2016)  Local site data  Local site data |

## Supplementary material 3: Sensitivity analyses

**Economic model 1**

***One-way sensitivity analyses (OWSA).*** With the first sensitivity analysis, the service delivery costs and all readmission costs were taken into consideration. To test the impact of the intervention involving shifting costs from non-elective to elective readmissions, we added a second sensitivity analysis where we separately explored elective readmissions costs (and service delivery costs) and non-elective readmissions costs (and service delivery costs). Additional univariate sensitivity analyses for the economic model included: bed days avoided for the comparator were increased to the upper limit 95% confidence interval (CI); the total costs of the comparator were equal to the lower limit 95% CI. In order to investigate the effect of the HHD schemes across time, we considered a follow-up period of three years to represent the medium-term follow-up period as reported by the Homelessness Monitor: England 2017. For the latter, the recommended discount rate of 3.5% was applied to both costs and benefits.

***Probabilistic sensitivity analysis (PSA).*** A non-parametric bootstrap technique was employed to explore the uncertainty of point estimates of the difference in mean 12-month costs and outcomes from the primary analyses. The results from bootstrap resampling were used to construct the 95% CI for incremental costs and the incremental bed days avoided, and to plot the cost-effectiveness plane and cost-effectiveness acceptability curve.

**Economic model 2**

The robustness of the economic model was tested using one-way deterministic sensitivity analyses looking at the following alternative scenarios: no service delivery; service delivery costs increased by 10%; service delivery costs increased by 20%; mean hospitalisation cost at follow-up =upper limit 95% CI; mean housing costs at follow up =upper limit 95% CI; and mean QALY at follow up =lower limit 95% CI.

In addition, probabilistic sensitivity analyses were undertaken and confidence intervals (CIs) for the cost per QALY gained were estimated using the non-parametric percentile bootstrap method. The data were re-sampled 1000 times to generate a mean cost and QALY gain from each point of interest and the resulting ICERs were calculated and plotted into the cost-utility plane. A cost-utility acceptability curve was also plotted.
